# Supplementary material for: Global Perspectives on Patient Safety: The Central Role of Nursing Management
Source: Healthcare (Basel). 2025 Dec 10;13(24):3240. doi: 10.3390/healthcare13243240 (PMC12733320; doi:10.3390/healthcare13243240)
Supplement: Supplementary file 1 [file healthcare-13-03240-s001.zip › Supplement_S1_Search_Strategy.pdf]

Supplementary File S1 – Search Strategy

Database: PubMed

Search Period: January 1, 2020 – April 30, 2025

Search String Used:

(("patient safety"[MeSH Terms] OR "patient safety"[Title/Abstract] OR "safety management"[MeSH Terms])
AND ("nursing management"[Title/Abstract] OR "nurse managers"[Title/Abstract] OR "nursing leadership"[Title/Abstract] OR "nursing administration"[Title/Abstract])
AND ("quality of care"[Title/Abstract] OR "healthcare quality"[Title/Abstract] OR "clinical governance"[Title/Abstract] OR "safety culture"[Title/Abstract])
AND ("hospital"[Title/Abstract] OR "health system"[Title/Abstract] OR "healthcare organization"[Title/Abstract]))
AND ("2020/01/01"[Date - Publication] : "2025/04/30"[Date - Publication])

Filters Applied:

- Publication Type: Peer-reviewed articles

- Language: English

- Species: Humans

Additional Notes:
This search strategy was adapted for use in CINAHL, Scopus, and Web of Science using equivalent controlled vocabulary and Boolean operators. Reference lists of included studies were also screened for additional relevant publications.
