# Supplementary material for: Global Perspectives on Patient Safety: The Central Role of Nursing Management
Source: Healthcare (Basel). 2025 Dec 10;13(24):3240. doi: 10.3390/healthcare13243240 (PMC12733320; doi:10.3390/healthcare13243240)
Supplement: Supplementary file 1 [file healthcare-13-03240-s001.zip › Supplement_S3_Completed_37_Studies.pdf]

Supplementary File S3. Summary of 37 Included Studies

This table lists the 37 peer-reviewed studies and global reports included in the narrative

Author / Year	Country / Region	Design / Method	Key Findings / Themes	Evidence Level (qualitative)
Author / Year	Country / Region	Design / Method	Key Findings / Themes	Evidence Level (qualitative)
Aiken et al., 2021	Chile / Latin America	Multilevel cross-sectional hospital study	Higher nurse staffing levels associated with lower mortality, fewer adverse events, and better patient ratings.	High
Laschinger et al., 2012	Canada	Cross-sectional survey of newly graduated nurses	Authentic leadership associated with lower bullying and burnout, and better retention outcomes.	Moderate
Alfadhalah et al., 2021	Kuwait	Cross-sectional safety-culture survey in primary care	Baseline assessment showed variation in safety-culture dimensions across primary health care centres.	Moderate
Alsaleh et al., 2025	Kuwait	Nationwide cross-sectional hospital survey	Differences in safety-culture perceptions between public and private hospitals with room for system-level improvement.	Moderate
Saleh et al., 2025	Saudi Arabia	Narrative/empirical review of national safety-culture implementation	Recent initiatives have expanded patient-safety culture programmes but gaps remain in	Moderate

			implementation and evaluation.	
Ball et al., 2014	United Kingdom	Cross-sectional survey of hospital nurses	Missed nursing care ('care left undone') strongly related to staffing levels and perceived quality of care.	High
Berger et al., 2023	Canada	Cross-sectional survey	Structural empowerment linked to more positive patient-safety climate among nurses.	Moderate
Boamah et al., 2018	Canada	Cross-sectional survey of acute-care nurses	Transformational leadership associated with higher job satisfaction and better reported patient-safety outcomes.	Moderate
Hamdan et al., 2024	Middle East (hospital settings)	Cross-sectional mediation analysis	Transformational leadership positively influences safety practices, mediated by patient-safety culture.	Moderate
Carayon et al., 2014	Primarily United States / conceptual	Conceptual and integrative review	Human factors and ergonomics approaches are central to designing safer systems and reducing error.	High
Insani et al., 2025	Various (systematic review)	Systematic review of economic evaluations	Digital health interventions can improve medication safety, though economic evidence is variable.	Moderate
Choi et al., 2020	Korea	Cross-sectional survey	Nurses frequently experience	Moderate

			patient-safety incidents, with under-reporting influenced by culture and workload.	
Cui et al., 2025	China	Failure Mode and Effects Analysis (FMEA) application	FMEA used to link safety-culture scores with adverse events and identify high-risk processes.	Moderate
Välimäki et al., 2024	Multiple countries	Mixed-methods systematic review	Evidence-based nursing leadership interventions associated with improved patient and workforce outcomes.	High
Donabedian, 1966	Conceptual / United States	Theoretical framework paper	Introduced the structure–process–outcome model, foundational for quality and safety evaluation.	High
Drennan & Ross, 2019	Global	Workforce and policy analysis	Global nurse shortages threaten care quality and safety, requiring coordinated policy responses.	Moderate
Welp et al., 2023	High-income settings	Survey-based analysis	Physician and nurse well-being closely linked to patient-safety perceptions; staff prefer systemic interventions.	Moderate
Adamuz et al., 2025	Hospitalized COVID-19 patients	Cross-sectional study	Nurse staffing coverage and care complexity factors associated with health outcomes	Moderate

			among COVID-19 inpatients.	
Galanis et al., 2021	Global	Systematic review and meta-analysis	High prevalence of nurse burnout during COVID-19, with implications for safety and retention.	High
Griffiths et al., 2019	United Kingdom	Retrospective longitudinal cohort study	Lower registered-nurse staffing associated with higher hospital mortality over time.	High
Havaei et al., 2021	Canada	Cross-sectional survey	Poor workplace conditions linked to worse nurse mental health during COVID-19.	Moderate
Alkubati et al., 2024	Critical-care settings	Cross-sectional survey of ICU nurses	Perceptions of patient-safety culture associated with reported adverse events in critical care.	Moderate
Kohn et al., 2000	United States	National report / policy analysis	Framed patient safety as a systems problem and called for comprehensive reforms to reduce errors.	High
Baines & Wheelock, 2021	Global / high-income focus	Scoping review	Nursing organizations play a key role in policy advocacy for safety and workforce issues.	Moderate
Hurtado-Arenas et al., 2024	Chile	Cross-sectional survey	Nurse-rated patient-safety culture in a Chilean hospital highlighted strengths and	Moderate

			improvement needs.	
McHugh et al., 2021	United States	Prospective observational study in two states	Nurse-to-patient ratio legislation associated with lower mortality, readmissions, and length of stay.	High
OECD, 2022	Global / OECD members	Economic and policy analysis	Investing in patient safety yields substantial returns and reduces avoidable harm costs.	High
Lucero et al., 2010	United States	Cross-sectional hospital study	Higher quality of nursing care associated with fewer adverse events in US hospitals.	Moderate
French et al., 2022	United States	Descriptive analysis of survey data	Pre-COVID conditions of nursing practice in hospitals and nursing homes revealed chronic staffing and resource issues.	Moderate
Sasso et al., 2021	Italy	Cross-sectional hospital survey	Missed nursing care associated with patient-safety indicators and management factors.	Moderate
Senek et al., 2020	United Kingdom	Cross-sectional survey	Care left undone associated with temporary nurse staffing ratios and workload.	Moderate
Abuosi et al., 2022	Ghana	Cross-sectional survey of healthcare facilities	Stronger safety culture linked to greater adverse-event reporting and learning orientation.	Moderate
Hesgrove et al., 2024	Hospital settings	Cross-sectional survey	Patient-safety culture positively	Moderate

			associated with workplace-safety culture among hospital staff.	
Bernardes et al., 2025	Hospital settings during COVID-19	Cross-sectional study	Authentic nursing leadership associated with a stronger safety climate across hospital units.	Moderate
Tencic & Roche, 2023	Acute-care hospitals	Cross-sectional study	Nurse–patient ratios linked to infection-control practices and safety outcomes.	Moderate
White et al., 2019	United States	Cross-sectional survey in nursing homes	RN burnout and job dissatisfaction associated with missed care in nursing homes.	Moderate
World Health Organization, 2020	Global	Global descriptive report	Highlighted global nursing workforce shortages and the need to invest in education, jobs, and leadership.	High
